# Expanding the clinical spectrum of interleukin-2 receptor alpha chain deficiency: two novel cases with long-term hematopoietic stem cell transplantation outcome and literature review

**DOI:** 10.3389/fimmu.2026.1716101

**Published:** 2026-01-23

**Authors:** Maha Alzubedy, Ahmed Sayed Osman, Huda Alajlan, Anas M Alazami, Hamoud Al-Mousa

**Affiliations:** 1 Section of Allergy and Immunology, Department of Pediatrics, King Faisal Specialist Hospital & Research Center, Riyadh, Saudi Arabia; 2 Department of Hematology Oncology, King Faisal Specialist Hospital & Research Center, Riyadh, Saudi Arabia; 3 Department of Translational Genomics, Genomic Medicine Centre of Excellence, King Faisal Specialist Hospital & Research Centre, Riyadh, Saudi Arabia; 4 College of Medicine, Alfaisal University, Riyadh, Saudi Arabia

**Keywords:** autoimmunity, CD25, hematopoietic stem cell transplantation, IL2RA, immune dysregulation, immunodeficiency, IPEX, regulatory T cells

## Abstract

**Background:**

Interleukin-2 receptor alpha chain (IL2RA, CD25) deficiency is a rare autosomal recessive inborn error of immunity characterized by profound immune dysregulation, susceptibility to infections, and autoimmunity. Only 13 cases of *IL2RA* deficiency have been reported worldwide since its first description in 1997, and experience with allogeneic hematopoietic stem cell transplantation (HSCT) for this condition remains very limited. This study aimed to describe the clinical, immunological, genetic, and HSCT outcomes of two siblings with *IL2RA* deficiency with a novel homozygous frameshift mutation and to review previously reported cases.

**Methods:**

The clinical course, laboratory findings, genetic diagnosis, and transplant outcomes of two patients managed at King Faisal Specialist Hospital & Research Centre were retrospectively reviewed. A comprehensive literature review was conducted to contextualize these cases.

**Results:**

Both patients presented with severe enteropathy, eczema, recurrent respiratory infections, growth failure, and features of allergic disease in early childhood. Immunological evaluation revealed hypergammaglobulinemia, impaired T-cell proliferation, reduced CD19^+^ B cells, inverted CD4/CD8 ratio, and absence of CD25 expression. Genetic analysis revealed a novel homozygous frameshift variant in *IL2RA* (c.166delC; p.R56fs). Both patients underwent HSCT with myeloablative conditioning. The younger sibling received marrow from a matched unrelated donor and achieved full donor chimerism with complete clinical and immunological recovery, remaining well 6 years after HSCT. The older sibling received marrow from a matched related donor and is alive and stable at 5 years of follow-up, with sustained donor chimerism and resolution of autoimmunity, complicated only by transient mild chronic graft-versus-host disease.

**Discussion:**

The two cases expand the mutational and clinical spectrum of *IL2RA* deficiency and provide long-term evidence that HSCT can cure immune dysregulation and susceptibility to infection. The findings underscore the importance of early genetic diagnosis and timely consideration of HSCT as definitive therapy for this rare but life-threatening disorder.

## 1 Introduction

The interleukin-2 receptor (IL-2R) plays a crucial role in T lymphocyte development, proliferation, and function. The IL-2R is a heterotrimeric complex composed of the IL-2Rα chain (CD25), the IL-2Rβ chain (CD122), and the common γ chain (CD132). Upon engagement with its ligand (IL-2), IL-2R initiates downstream signaling pathways critical for immune regulation, including the JAK1/3-STAT5 and PI3K-AKT-mTOR pathways, which are essential for T-cell proliferation, survival, and differentiation (1, 2).

Among the receptor subunits, IL-2Rα (CD25) is unique in that it does not transduce signals independently. CD25 binds IL-2 with low affinity on its own. However, it markedly enhances receptor avidity when co-expressed with CD122 and CD132, thereby forming the high-affinity IL-2 receptor complex (3, 4). CD25 is transiently upregulated on activated conventional T cells and constitutively expressed at high levels on CD4^+^ regulatory T cells (Tregs), where it is essential for IL-2-mediated signaling required for their development, expansion, and suppressive function (3, 5). Through these mechanisms, IL-2 signaling via CD25 plays a non-redundant role in maintaining peripheral immune tolerance and preventing autoimmunity.

Loss-of-function mutations in IL-2R alpha chain (*IL2RA*), the gene encoding CD25, result in CD25 deficiency, a rare autosomal recessive primary immunodeficiency disorder, which was first described in 1997 (1). Since its discovery, only 13 cases have been reported in the literature. This disorder is characterized by defective Treg cell homeostasis and function, which leads to profound immune dysregulation. Affected patients often present with an array of symptoms in infancy or early childhood, including enteropathy, severe eczema, autoimmune cytopenias, type 1 diabetes mellitus, thyroiditis, lymphadenopathy, hepatosplenomegaly, and recurrent infections. Furthermore, the clinical phenotype frequently overlaps with Immune dysregulation, polyendocrinopathy, enteropathy, X-linked (IPEX) syndrome, caused by FOXP3 mutations, reflecting their shared mechanism of Treg dysfunction. Typically, immunologic investigations reveal normal or elevated total T and B cell counts, elevated serum IgG and IgA levels, impaired T-cell proliferation in response to mitogens, and near absence of CD25 expression on CD4^+^ T cells. Flow cytometry plays a crucial role in confirming the absence of CD25 surface expression, whereas molecular analysis confirms the presence of biallelic pathogenic variants in *IL2RA* (1, 3–11). Despite supportive therapies, including immunosuppression and infection prophylaxis, poor clinical outcomes are frequently observed due to progressive immune-mediated tissue damage and infectious complications. Hematopoietic stem cell transplantation (HSCT) remains the only curative treatment that aims to restore normal immune function by reconstituting a healthy Treg compartment. However, reports on HSCT in CD25 deficiency are very limited. Only two cases with successful long-term follow-up after transplantation have been described in the literature (1, 4). This study aimed to present the clinical, immunological, genetic, and transplantation outcomes of two siblings from a consanguineous family diagnosed with CD25 deficiency due to a novel homozygous mutation in *IL2RA*. Both patients manifested severe immune dysregulation and underwent successful HSCT. This study adds to the limited body of literature on this rare disorder and provides further support for early definitive therapy through allogeneic HSCT in patients with *IL2RA* deficiency.

## 2 Materials and methods

This was a retrospective chart review of two patients with genetically confirmed *IL2RA* deficiency who were followed up at the Immunodeficiency Clinics of King Faisal Specialist Hospital and Research Centre (KFSHRC), Riyadh, Saudi Arabia. Clinical, immunological, and genetic characteristics were evaluated, as well as HSCT outcomes and immune reconstitution at the most recent follow-up. This study was approved by the Institutional Review Board of KFSHRC. Written informed consent was obtained from the patients or their legal guardians for the publication of potentially identifiable data or images.

### 2.1 Cellular and immunological assays

Peripheral blood leukocyte subsets were analyzed using immunofluorescence staining and flow cytometry. Monoclonal antibodies specific for T cells (CD3, CD4, and CD8), natural killer (NK) cells (CD16 and CD56), and B cells (CD19) were used (Becton, Dickinson & Co., San Jose, CA, USA). T-cell functional capacity was evaluated *in vitro* by measuring proliferative responses to phytohemagglutinin stimulation. Serum immunoglobulin (Ig) levels, including IgG, IgA, and IgM, were quantified using nephelometry.

### 2.2 Genetic testing

Genomic DNA was extracted from peripheral blood samples collected from patients and their parents during routine clinical practice. Next-generation sequencing was performed using a targeted primary immunodeficiency panel. Sequence reads were aligned with the human reference genome (GenBank). Variants were identified and analyzed as previously described (12). The Saudi Human Genome Database was consulted to exclude unreported population-specific single-nucleotide polymorphisms. The pathogenicity of the identified missense variants was assessed using *in silico* prediction tools.

### 2.3 Flow cytometry

Carboxyfluorescein succinimidyl ester (CFSE) T-cell proliferation assay was performed. Thawed peripheral blood mononuclear cells (PBMCs) were rested in complete medium for 2 h and then stained with 1 µM CFSE (BD) at 37°C for 15 min. After extensive washing, cells were seeded in a U-bottom 96-well plate at a density of 1 × 10^6^ cells/ml. Stimulation was performed using Dynabeads Human T-Activator CD3/CD28 (Thermo Fisher) supplemented with IL-2 (20 units/ml, R&D Systems) for 3 days before analysis by fluorescence-activated cell sorting in the presence of 4′,6-diamidino-2-phenylindol (DAPI) for live/dead cell discrimination.

Thawed and rested PBMCs were seeded and stimulated to detect cell surface markers. The cells were stained at the appropriate time points (days 0, 1, and 3) with the following antibodies: anti-CD4-FITC, anti-CD25-PE-Cy7, and anti-CD69-APC (all from BD). The cells were then assessed by fluorescence-activated cell sorting in the presence of DAPI.

### 2.4 Preparative regimen, transplantation, and supportive care

Both patients underwent HSCT. Human leukocyte antigen (HLA) compatibility was determined by high-resolution molecular typing of class I (HLA-A, HLA-B, and HLA-C) and II (HLA-DRB1 and HLA-DQB1) loci. Stem cell sources included unmanipulated bone marrow from either an HLA-matched sibling or a matched unrelated donor. The conditioning regimen included busulfan, fludarabine, and antithymocyte globulin (ATG). Chimerism was monitored using short tandem repeat analysis.

## 3 Results

### 3.1 Patient 1

An 11-year-old male patient, the youngest of two siblings born to consanguineous Saudi parents, was referred to our center due to a complex clinical history of chronic diarrhea, recurrent respiratory infections, severe atopic dermatitis, and progressive growth failure. His symptoms began in early infancy and persisted despite multiple therapeutic interventions. At the age of 8 years, the patient experienced frequent episodes of otitis media and lower respiratory tract infections, which often required antibiotics. Chest high-resolution computed tomography showed bilateral bronchiectasis, indicative of chronic pulmonary damage. The patient had additional atopic features, including persistent bronchial asthma, allergic rhinitis, and adenoidal hypertrophy, for which adenotonsillectomy was performed at the age of 7 years.

Despite a normal appetite, his growth remained severely delayed, with a height consistently below the third percentile. Endocrinologic evaluation revealed generalized osteopenia and short stature unresponsive to growth hormone therapy, along with iron deficiency anemia. Gastrointestinal evaluation, including upper and lower endoscopy with biopsies were normal. At the age of 9 years, he developed painless generalized lymphadenopathy. Imaging revealed bilateral hilar lymph node calcifications. However, the extensive infectious workup, including microbiological cultures from axillary lymph nodes biopsy and PCR-based testing for tuberculosis, CMV, and Epstein–Barr virus (EBV) was negative.

**Figure 1:**
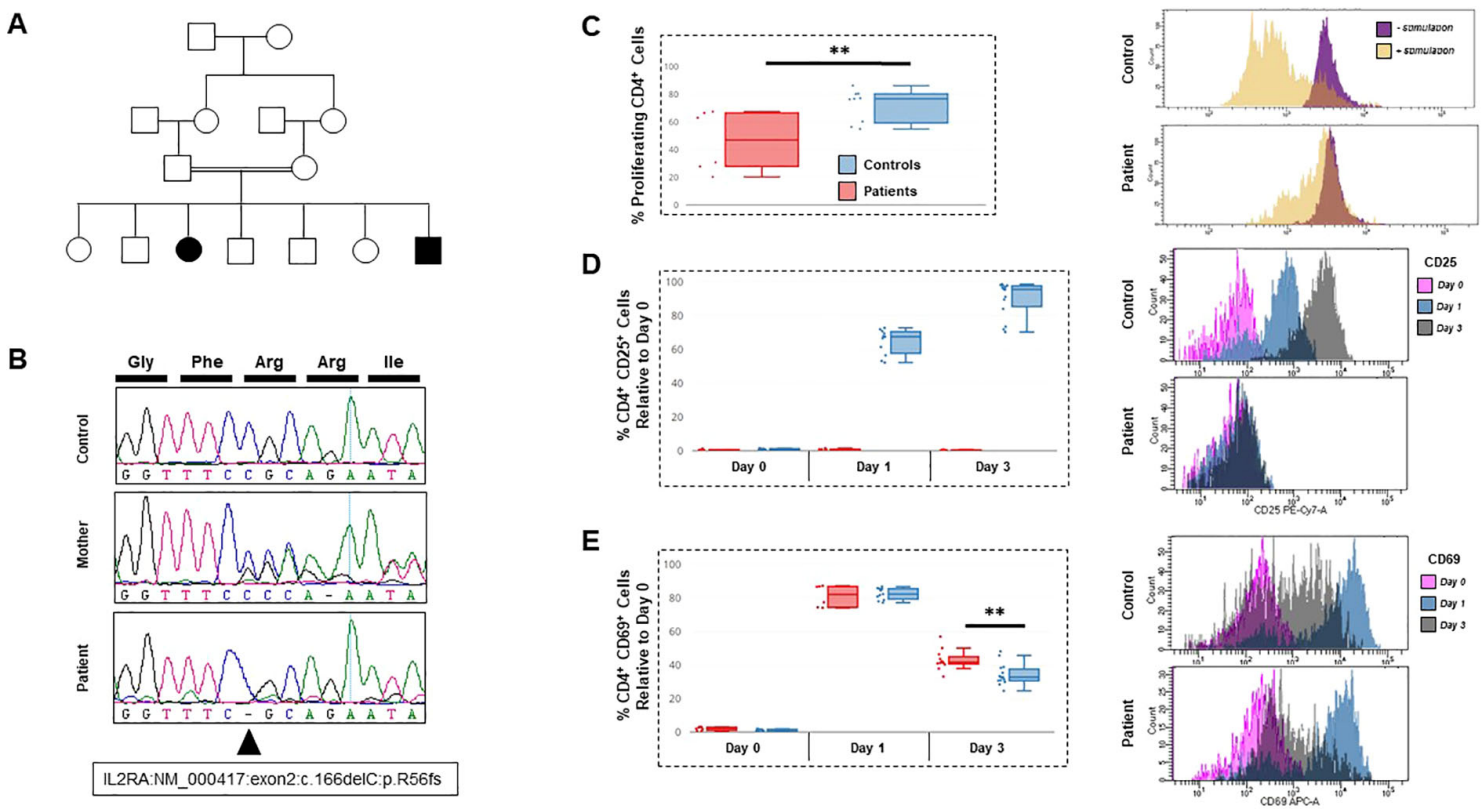
Molecular and cellular characterization of two siblings presenting with CD25 deficiency. **(A)** Pedigree of the family reported in this study. **(B)** Sanger sequencing chromatograms for IL2RA (c.166delC) from patient, parent, and control samples. Arrowhead denotes the position of the frameshift variant, and amino acid codons for the wildtype sequence are provided at the top. **(C)** Left: Box-and-whiskers data indicating T cell proliferation from the patient PBMCs, versus three healthy controls. CFSE-stained cells were stimulated for 3 days then gated on viable (DAPI-) CD4+ T helper cells. Right: representative CFSE histograms of one healthy control and one patient, with and without stimulation. **(D)** Left: summary data indicating cell surface CD25 expression on viable CD4+ T cells, prior to stimulation (day 0) and at days 1 and 3 post-stimulation. Data include both patients along with five healthy controls. Right: representative histograms for one patient and one control, with color-coding to show progression of the peaks over the time course of the experiment. **(E)** Left: summary data for CD69 cell surface expression, for the same cells analyzed in the previous panel. Right: representative histograms for one patient and one control. For all experiments, individuals were assessed at least as independent triplicates. Asterisks indicate significance levels (**p < 0.01; unpaired Student’s t-test).

The initial immunological assessment revealed normal total leukocyte and lymphocyte counts. However, T-cell phenotyping revealed a persistently inverted CD4^+^/CD8^+^ ratio. T-cell proliferation in response to mitogens (phytohemagglutinin, anti-CD3) was markedly reduced. CD19^+^ B cell counts were slightly decreased, and NK cell numbers were within age-matched reference ranges. Ig profiling revealed hypergammaglobulinemia with elevated IgG and IgA levels and normal IgM and IgE levels. Antibody responses to tetanus toxoid and pneumococcal polysaccharides were preserved. Molecular analysis using the next-generation sequencing primary immunodeficiency panel revealed a novel homozygous frameshift mutation in *IL2RA* (c.166delC; p.R56fs), which was confirmed by Sanger sequencing. Both parents were heterozygous carriers. Family pedigree and Sanger sequencing chromatograms for IL2RA (c.166delC) from patient, parent, and control samples is shown (**Figures 1A, B**). Flow cytometry confirmed the absence of CD25 surface expression and significant impairment of T-cell proliferation in response to Dynabeads Human T-Activator CD3/CD28 (**Figures 1C, D**). Patient and control CD4^+^ cells reached similarly high levels of CD69 surface expression within 24 h of stimulation. Interestingly, the patterns had diverged by day 3. Control cells displayed a broad spectrum of CD69 depletion, with many cells losing expression entirely, whereas patient cells showed a much narrower, peaked distribution, with almost no cells lacking surface CD69. This resulted in a significantly reduced depletion of surface CD69 in the patient cells by day 3 (**Figure 1E**). Monthly IVIG replacement therapy and *Pneumocystis jirovecii* prophylactic antibiotics were initiated. The patient underwent allogeneic HSCT at the age of 12 years due to ongoing infections, poor quality of life, and progressive disease manifestations. The donor was a 10/10 HLA-matched unrelated individual. He received 10 × 10^6^ CD34^+^ cells/kg. GvHD prophylaxis consisted of cyclosporine and ATG. The patient’s early post-transplant course was complicated by grade II acute cutaneous GvHD, which responded rapidly to systemic corticosteroids and mycophenolate mofetil. Additionally, the patient developed CMV viremia, which was effectively managed with antiviral therapy. Chimerism studies demonstrated full donor myeloid engraftment and 95% donor T-cell chimerism by day +40. Diarrhea, eczema, and infections showed complete resolution. Ig replacement was stopped 11 months after HSCT, after which revaccination was initiated with protective vaccine responses and marked improvement in growth parameters. His laboratory finding pre and post HSCT are summarized on **Supplementary Table 1**. At his most recent follow-up at 18 years of age (6 years after HSCT), the patient remained clinically stable, with complete donor chimerism, restored T-cell function, and no evidence of autoimmune disease.

### 3.2 Patient 2

A 19-year-old female patient, the older sibling of Patient 1, presented with a parallel but progressively worsening clinical course. She experienced chronic eczema involving more than 70% of her body surface area, persistent diarrhea, failure to thrive, and multiple food allergies in her early childhood. At the age of 8 years, she began to experience recurrent sinopulmonary infections, which often required hospital-based management. Despite adequate oral intake, she exhibited poor weight gain and linear growth, with her body mass index persistently falling below the fifth percentile. Additionally, she developed allergic disease features, including asthma, allergic rhinitis, and chronic urticaria episodes. Her eczema was complicated by recurrent skin infections, which further contributed to her morbidity.

The initial laboratory evaluation revealed normal total leukocyte and lymphocyte counts. However, she had microcytic anemia. Immunophenotyping showed a marked reduction in CD19^+^ B cell count (50/mm³), whereas CD4^+^ (779/mm³), CD8^+^ (495/mm³), and NK (CD56^+^CD16^+^, 153/mm³) cell subsets were within normal age-matched reference ranges. Serum Ig analysis revealed elevated IgG and IgA levels with normal IgM concentration. Functional antibody testing showed suboptimal responses to protein antigens. However, adequate responses to polysaccharide antigens were observed. Genetic testing confirmed the presence of the same novel homozygous frameshift mutation in *IL2RA* (c.166delC; p.R56fs) as that in her younger brother.

The patient was initially conservatively managed for 1 year with nutritional support, *Pneumocystis jirovecii* infection prophylaxis, and dermatologic care consisted primarily of topical corticosteroids and emollients. However, at the age of 21 years, she experienced increasing clinical deterioration, including more frequent hospitalizations due to lower respiratory tract infections and widespread skin infections. Given her worsening trajectory and genetic confirmation of IL2RA deficiency, a decision to perform HSCT was made. She underwent HSCT from a 10/10 HLA-matched related donor. She received 7.31 × 10^6^/kg CD34^+^ hematopoietic stem cells. GvHD prophylaxis consisted of tacrolimus and methotrexate. The early post-transplant course was notable for grade I mucositis and febrile neutropenia, which were conservatively managed. Neutrophil engraftment was achieved on day +16. She developed grade II acute cutaneous GvHD on day +20, which responded well to topical and systemic corticosteroids. On day +34, donor chimerism analysis demonstrated complete (100%) engraftment. Additionally, she developed CMV viremia, mild transaminitis, and diarrhea, which resolved with appropriate antiviral and supportive therapy.

The patient developed mild chronic musculoskeletal GvHD with proximal muscle weakness and arthralgia approximately 18 months after HSCT. She was treated with imatinib and temporary reintroduction of tacrolimus, which gradually improved her symptoms. At her most recent evaluation, 5 years after HSCT, the patient remained in stable clinical condition with 100% donor chimerism, normalized gastrointestinal function, eczema and recurrent infection resolution, and no active signs of autoimmune or GvHD-related complications. Her laboratory finding pre and post HSCT are summarized on **Supplementary Table 1**.

## 4 Discussion

*IL2RA* (CD25) deficiency is a rare autosomal recessive inborn error of immunity. This disorder belongs to a broader category of diseases of Treg biology, which includes *FOXP3* deficiency (IPEX syndrome), *STAT5b* deficiency, and *STAT1*/*STAT3* gain-of-function disorders. These entities share the features of autoimmunity, endocrinopathies, and enteropathy. However, *IL2RA* deficiency is unique in that it combines opportunistic susceptibility to infection with severe autoimmunity, reflecting the dual requirement of IL-2 signaling for effector immunity and tolerance (13). Since the first description of *IL2RA* deficiency in 1997, only 15 patients, including our two novel cases, have been reported worldwide. These cases provide valuable insights into the non-redundant role of IL-2 signaling in maintaining human immune homeostasis (1, 3–11, 15).

**Table 1** summarizes the clinical, immunological, and genetic characteristics of all reported cases of *IL2RA* deficiency. The clinical presentation of *IL2RA* deficiency is heterogeneous but recurring. Patients typically present with opportunistic and recurrent infections, such as CMV, EBV, *Candida*, adenovirus, *Pseudomonas*, and *Klebsiella*, early in life. Chronic diarrhea, villous atrophy, and failure to thrive are frequent features that mimic other forms of combined immunodeficiency. Autoimmunity is significantly prevalent. The spectrum includes autoimmune hemolytic anemia, immune thrombocytopenia, autoimmune neutropenia, type 1 diabetes mellitus, autoimmune thyroiditis, hypothyroidism, hyperparathyroidism, and autoimmune hepatitis. Additional immune-mediated manifestations include enteropathy, dermatitis, alopecia, bullous pemphigoid, granulomatous hepatitis, and pulmonary hemorrhage. These features reflect the breakdown of the central and peripheral tolerance. Hepatosplenomegaly, lymphadenopathy, asthma, short stature, and chronic inflammatory lesions are other systemic findings. The dual burden of infections and autoimmunity distinguishes *IL2RA* deficiency from other combined immunodeficiencies, aligning it more closely with regulatory T-cell (Treg) biology disorders.

**Table 1:** Clinical, immunological, and genetic characteristics and outcome of HSCT of reported *IL2RA* (CD25) deficiency patients.

Patient/sex	Age at onset	Age at diagnosis	Infections	Autoimmunity/endocrinopathies	Other clinical features	Immunological findings	Mutation	Therapy	HSCT	Date of report (reference)
P1/M	6 months		Cytomegalovirus (CMV) pneumonitis, *Candida* esophagitis, and adenovirus gastroenteritis	No	Chronic diarrhea, FTT, oral thrush, lymphadenopathy, hepatosplenomegaly, chronic mandibular inflammation, and chronic lung disease	High IgG, low IgA, low CD4^+^, abnormal CD4/CD8 ratio of 1:1, impaired mitogen response, and absent of CD25 expression	Homozygous c.60-64del; p.21fs	NA	Yes (alive)	1997 (1)
P2/M	6 weeks	8 years	CMV pneumonitis and EBV	Severe AIHA, neutropenia, hypothyroidism, and IDDM	Chronic diarrhea, autoimmune enteropathy, recurrent sinopulmonary infections, eczema, systemic lymphadenopathy, and hepatosplenomegaly	High IgG, normal IgA, normal IgM, and absence of CD25 expression upon CD4 activation	Compound heterozygous c.301C>T; c.693insA; p.101stop; p.232fs	Steroid, rituximab, IVIG, and cyclosporine A	No	2007 (3)
P3/F	1 month	8 years	CMV	Autoimmune thyroiditis, alopecia universalis, and psoriasiform dermatitis	Chronic diarrhea, autoimmune enteropathy, villous atrophy, bullous pemphigoid, cellulitis, and multiple lymphadenopathies	Normal IgG and IgM, elevated IgA and IgE, inverted CD4/CD8, low B/NK, and absence of CD25 expression upon CD4 activation	Homozygous c.497G>A; p.S166N	Steroids, tacrolimus, methotrexate, mycophenolate mofetil, and rapamycin	No	2013 (6)
P4/F	6 days	5 years	Severe varicella	Alopecia	Severe atopic dermatitis, chronic diarrhea, several respiratory infections, asthma, dysmorphic features, and follicular bronchiolitis with lymphocyte hyperplasia	High IgG, absence of IgG4, normal IgA and IgM, impaired specific polysaccharide response, and low Tregs	Homozygous c.122A>C; p.Y41S	Steroid, antibiotic prophylaxis, rapamycin, and IVIG	No	2013 (5)
P5/M	10 days	10 months	CMV disease	Autoimmune hepatitis, AIHA, neutropenia, IDDM, and autoimmune thyroiditis	Pulmonary hemorrhage, thrombocytopenia, and hepatosplenomegaly	Normal lymphocytes and Ig levels and absence of CD25 expression	Homozygous c.418T>C; p.Tyr140His	Steroids, cyclophosphamide, rituximab, and mycophenolate mofetil	No	2014 (15)
P6/F	3 years	12 years		Hyperthyroidism and alopecia universalis	Eczema, chronic diarrhea, hepatosplenomegaly, lymphadenopathy, and severe dry eyes	Absence of CD25 expression	NA	Steroid and cyclosporine A	NA	2016 (9)
P7/M	NA	NA	NA	IDDM		Anti-GAD and ICA512	Compound heterozygous c.530A>G c.800delA	NA	NA	2017 (7)
P8/M	NA	NA	NA	Diabetes autoantibodies	Ear infections and eczema	Anti-GAD, MIAA, and ICA	Compound heterozygous c.530A>G c.800delA	NA	NA	2017 (7)
P9/F	NA	NA	NA	Immune thrombocytopenic purpura and autoimmune neutropenia	Ear infections, hemolytic anemia, nummular dermatitis, hypercellular bone marrow, and mouth ulcer	Inverted CD4/CD8 ratio and antiplatelet	Compound heterozygous c.530A>G c.800delA	NA	NA	2017 (7)
P10/M	1 months	2 months	Pseudomona, HHV-6 in stool	No	Chronic diarrhea, villous atrophy, and FTT	Normal IgG, IgA, and IgM, high IgE, low naïve T cells, low CD4/CD8, low B cells, and absence of CD25 expression	Homozygous c.151T>G; p.Cys51Gly	NA	Yes (2x) (alive)	2019 (4)
P11/F	2 months	9 years	*Aspergillus* infection	AIHA and IDDM	Recurrent respiratory infection, chronic diarrhea, hepatosplenomegaly, lymphadenopathy, and bronchiectasis	ANA, pANCA, and cANCA positive and absence of CD25 expression	Compound heterozygous c.64G>A p.Ala21Serfs*26 c.340C>T p.Gln114Ter	Steroid, IVIG, and rapamycin	No	2019 (10)
P12/M	7 months	12 years	*Klebsiella pneumonia* urinary tract infection	No	Chronic diarrhea, autoimmune enteropathy, villous atrophy, eczema, granulomatous hepatitis, hepatosplenomegaly, lymphadenopathy, pancolitis, and death	High IgG, IgA, and IgM	Homozygous c.504 C>A; p.Cys168Ter	NA	No	2022 (8)
P13/M	1 month	7 months	–	Autoimmune enteropathy	Chronic diarrhea, metabolic acidosis, and eczema	Normal IgG, IgA, and IgM and normal lymphocyte subsets	Homozygous c.64 + 1G > A	Steroid	No	2022 (11)
P14/M (our case)	1 year		MRSA otitis, and rhinovirus	Chronic diarrhea	Asthma, short stature, atopic dermatitis and recurrent sinopulmonary infections	Low CD4/CD8 ratio, low CD19^+^, impaired T-cell response, and high IgG/IgA	Homozygous c.166delC; p.R56fs	IVIG, prophylactic antibiotics	Yes (alive)	Present study
P15/F (our case)	3 years		*Morganella* Urinary tract infections	AIHA and hyperparathyroidism	FTT and recurrent sinopulmonary infections	Very low CD19^+^, high IgG/IgA, and poor antibody responses	Homozygous c.166delC; p.R56fs	IVIG, prophylactic antibiotics	Yes (alive)	Present study

HSCT, Hematopoietic Stem Cell Transplantation; CMV, Cytomegalovirus; EBV, Epstein-Barr virus; FTT, Failure to thrive; AIHA, Autoimmune hemolytic anemia; IDDM, Insulin dependent diabetes mellitus; IVIG, Intravenous immunoglobulin; HHV-6, Human herpes virus-6; NA, Not available.

The absence of or marked reduction of CD25 expression on activated CD4+ T cells is the immunological hallmark of *IL2RA* deficiency, as observed in our patients. Functionally, this disrupts IL-2-mediated signaling, thereby impairing effector T-cell proliferation and Treg differentiation. CD69 expression was similarly induced in our patient and control CD4^+^ T cells at 24 hours after CD3/CD28 stimulation, indicating preserved early activation. However, by day 3, patient cells demonstrated significantly impaired down-modulation of CD69, suggesting altered activation kinetics rather than defective TCR engagement. In the context of IL2RA deficiency, this abnormal CD69 regulation likely reflects disrupted IL-2–dependent signaling required for activation resolution and immune homeostasis. An abnormal CD4/CD8 ratio (often inverted), reduced numbers of naïve T cells, and decreased numbers of circulating B cells (CD19^+^) are common findings. Ig levels vary. IgG and IgA levels are often elevated, IgM levels may be normal or low, and IgE levels are sometimes markedly increased. Antibody responses to polysaccharide and protein antigens are frequently impaired. These abnormalities are correlated with defective IL-2-driven expansion of antigen-specific T cells and insufficient Treg control. The clinical and immunological phenotype closely parallels murine Il2ra−/− models, which develop lymphoproliferation, multi-organ autoimmunity, and premature death, highlighting the conserved role of IL-2/CD25 signaling in immune tolerance (14).

Pathogenic variants in *IL2RA* include biallelic missense, nonsense, frameshift, and splice site mutations. These variants invariably abolish or severely impair CD25 expression and function. Despite phenotypic variability, no clear genotype–phenotype correlation has been observed, reflecting the common downstream consequence of defective IL-2 receptor signaling.

Before definitive therapy, the management was based on immunosuppressive and supportive modalities, such as corticosteroids, cyclosporine, tacrolimus, mycophenolate mofetil, rapamycin, rituximab, cyclophosphamide, and IVIG. Although they can transiently control autoimmunity, they fail to prevent disease progression. Several patients have been reported to succumb to infections or uncontrolled immune dysregulation despite prolonged therapy. At least four patients with *IL2RA* deficiency have undergone HSCT successfully to date, including our two cases. All transplanted cases are alive, and infections and autoimmunity were resolved, confirming that HSCT is a curative treatment for this disorder. The first reported case of IL2RA deficiency successfully underwent transplantation. However, full details of the conditioning regimen and immune reconstitution were not provided (1). One patient required two transplants. In the first transplant, a haploidentical allogeneic HSCT was performed at 3 months of age using TCRα/β and CD19 depletion, a reduced-intensity conditioning regimen consisting of treosulfan, fludarabine, and ATG, and GvHD prophylaxis consisting of anti-CD20. This transplant failed to achieve engraftment. In the second transplant, HSCT from a matched unrelated donor was performed at 7 months of age using a reduced-intensity conditioning regimen consisting of low-dose busulfan, fludarabine, and ATG and GvHD prophylaxis consisting of methotrexate and cyclosporine. This transplant was successful, with stable donor chimerism (95% donor) at 4 months after HSCT, robust immune reconstitution, and complete symptom resolution. The patient developed mild skin GvHD, which responded well to immunosuppressive therapy, underscoring the potential challenges of immune recovery after transplantation (4).

The results of our two cases confirm the feasibility and effectiveness of HSCT in curing *IL2RA* deficiency, even when performed at an older age and with a myeloablative regimen to ensure stable engraftment and durable immune reconstitution. These findings indicate that although early transplantation is ideal to prevent irreversible complications, HSCT in patients diagnosed later in the disease course can still achieve favorable outcomes. The successful correction of immune dysregulation and resolution of autoimmunity after HSCT highlight the crucial role of IL-2/IL2RA signaling in maintaining immune tolerance.

This study has some limitations that warrant consideration. First, regulatory T-cell (Treg) enumeration was not performed for both patients, this could have provided additional insight into the extent of Treg deficiency and its restoration following hematopoietic stem cell transplantation. Nevertheless, several lines of evidence strongly support the presence of profound Treg dysfunction in our patients. Our patients demonstrated complete absence of CD25 surface expression on activated CD4^+^ T cells, a defining molecular and functional hallmark of IL2RA deficiency. In addition, impaired IL-2–dependent T-cell proliferation, abnormal activation kinetics, and severe early-onset immune dysregulation with enteropathy, eczema, and autoimmunity are highly characteristic of defective Treg biology. Prior studies have consistently shown that IL2RA mutations lead to impaired Treg development and function despite variable absolute Treg numbers, indicating that functional impairment rather than numerical deficiency is central to disease pathogenesis. Second, the small number of patients, retrospective design, and relatively short follow-up periods across the global literature limit the generalizability of findings and preclude definitive assessment of long-term risks, such as chronic GvHD, secondary malignancies, and late infections. Third, our patients underwent transplantation in heterogeneous clinical contexts, thereby limiting the ability to define a single optimal HSCT approach. Despite these limitations, this study has some notable strengths. This is the first study to present cases of *IL2RA* deficiency from the Middle East. This study contributes two novel genetically confirmed patients to the global literature and provides detailed clinical, genetic, and transplant data. Our patients underwent transplantation with myeloablative conditioning regimens and were followed up for a relatively longer duration compared with previously reported cases, thereby providing valuable insights into the durability of engraftment, immune reconstitution, and sustained resolution of autoimmunity. These findings expand the clinical spectrum of *IL2RA* deficiency and support the use of HSCT as a curative option, even when performed beyond infancy.

The findings of this study and those of previously reported cases highlight the need for timely genetic diagnosis in children presenting with early-onset autoimmunity and recurrent infections, as *IL2RA* deficiency may be under recognized in this context. Early identification allows for informed counseling of families and timely referral for transplantation before the development of severe, irreversible organ damage. Furthermore, future collaborative efforts and long-term follow-up of patients with transplantation are essential for better defining the optimal conditioning regimens, strategies to minimize GvHD, and predictors of durable immune reconstitution in this rare but informative disorder.

## 5 Conclusion

*IL2RA* deficiency highlights the crucial role of IL-2/CD25 signaling in regulating immune responses and maintaining tolerance. This diagnosis should be suspected in infants and children presenting with an unusual combination of opportunistic infections and multi-organ autoimmunity, particularly autoimmune cytopenias and endocrinopathies. Currently, HSCT is the only definitive therapy and should be strongly considered once the diagnosis is confirmed. Given the mechanistic clarity of the defect, future studies should explore gene therapy or adoptive Treg transfer. Long-term follow-up of patients with transplantation is crucial to assessing the durability of immune reconstitution, relapse of autoimmunity, and quality of life outcomes.

## Data Availability

The data presented in this study are deposited in the ClinVar repository, accession number SCV006550851, and can be accessed at https://www.ncbi.nlm.nih.gov/clinvar/.
