# Supplementary material for: Expanding the clinical spectrum of interleukin-2 receptor alpha chain deficiency: two novel cases with long-term hematopoietic stem cell transplantation outcome and literature review
Source: Front Immunol. 2026 Jan 23;17:1716101. doi: 10.3389/fimmu.2026.1716101 (PMC12876145; doi:10.3389/fimmu.2026.1716101)
Supplement: Supplementary file 1 [file Table1.docx]

Supplementary table 1: Laboratory characteristics pre and post HSCT

|  | Patient 1 | | Patient 2 | |
| --- | --- | --- | --- | --- |
|  | Pre-HSCT  (age 10 years) | 7- year post-HSCT  (age 20 years) | Pre-HSCT  (age 20 years) | 5 years post-HSCT  (age 27 years) |
| CD3/mm^3^ | 2243 | 2247 | 1331 | 3393 |
| CD4/mm^3^ | 851 | 1163 | 779 | 1327 |
| CD8/mm^3^ | 1146 | 906 | 498 | 1972 |
| CD19/mm^3^ | 159 | 489 | 50 | 529 |
| CD15/56/mm^3^ | 322 | 380 | 153 | 187 |
| CD3 45RA% | 31 | 40 | 31 | ND |
| CD4 45RA% | 1 | 17 | 6 | ND |
| CD3 45RO% | 42 | 23 | 54 | ND |
| CD4 45RO% | 31 | 10 | 46 | ND |
| IgG (g/L) | 16.1 | 10.2 | 13.7 | 10.7 |
| IgM (g/L) | 2.08 | 0.62 | 6.85 | 2.05 |
| IgA (g/L) | 7.83 | 3.68 | 1.21 | 0.86 |
| IgE (KU/L) | 17 | ND | ND | ND |
| Tetanus toxoid IgG (mg/dl) | 8.16 | >119 | 3.29 | ND |
| Pneumococcal Polysaccharide IgG (mg/dl) | 45.28 | 50.1 | 22.8 | ND |
| PHA response CPM (RR%) | 17330 (14%) | ND | 83368 (58%) | ND |
| Donor lymphoid cell chimerism % | - | 100 | - | 91 |
| Donor myeloid cell chimerism % | - | 100 | - | 100 |

Normal reference values: [CD3: 6–13 years: (1700–1900 per mm3),18-44 years: (782-2834), CD4: 6–13 years: (800–1700 per mm3), 18-44 years: (322-1750) CD8: 6–13 years: (700– 1000 per mm3), 18-44 years: (338-1086), CD19: 6–13 years: (400–800 per mm3), 18-44 years: (67-555), CD16+ 56+: 6–13 years: 200–400 per mm3), IgG (12 years- adult): 7-16 g/L, IgA: 0.7– 4 g/L, IgM: 0.4–2.3 g/L, and IgE: 5-500 KU/L

*ND* not done
